# Incidental “Urinoma” in ^18^F-FDG PET/CT Scan

**DOI:** 10.4274/mirt.galenos.2018.14632

**Published:** 2019-09-06

**Authors:** Aziz Gültekin, Olga Yaylalı, Tarık Şengöz, Doğangün Yüksel

**Affiliations:** 1 Pamukkale University Faculty of Medicine, Department of Nuclear Medicine, Denizli, Turkey

**Keywords:** Urothelial carcinoma, urinoma, positron emission tomography

## Abstract

^18^F-FDG PET/CT scanning was performed for the primary staging of a 47-year-old man with urothelial carcinoma. The patient underwent biopsy by ureteroscopy 15 days ago and the PET images revealed ^18^F-FDG accumulation in the right retroperitenal region, compatible with an “urinoma”.

## Abstract (tr)

Kırk yedi yaşındaki ürotelyal kanserli erkek hastaya primer evreleme için ^18^F-FDG PET/BT görüntüleme yapıldı. ^18^F-FDG PET/BT görüntülemesinden 15 gün önce üreteroskopi ile biyopsi yapılan hastanın sağ retroperitenal bölgesinde “ürinoma” ile uyumlu ^18^F-FDG birikimi gözlendi.

## Figures and Tables

**Figure 1:**
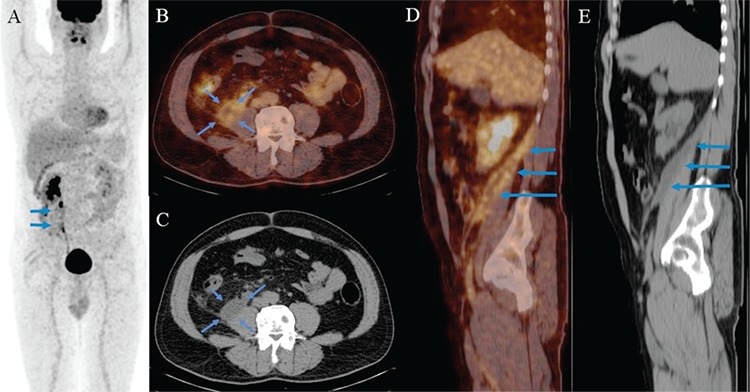
A 47-year-old man with a diagnosis of urothelial carcinoma was referred for primary staging with ^18^F-FDG PET/CT scan. The ^18^F-FDG PET/ CT scanning was performed 15 days after ureteroscopic biopsy. Imaging was performed 60 minutes after I.V. injection of 395 MBq ^18^F-FDG, on an integrated 16 slice PET/CT, with scanning from the skull base to the knee. ^18^F-FDG PET/CT images [(A) MIP; (B) axial fusion; (C) axial CT; (D) sagittal fusion; (E) sagittal CT) showed tracer accumulation in the fluid collection extending to the pelvis along the retroperitoneal area (SUV_max_: 4.90, mean density; 1 Hounsfield units), associated with the right ureter, in the right iliopsoas region adjacent to the muscle (A-E blue arrows).

**Figure 2:**
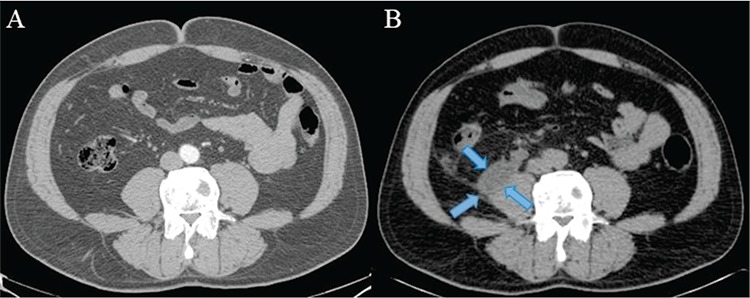
Contrast enhanced CT image before the biopsy (A) and CT image of PET/CT after the biopsy (B). Urinoma was shown (B blue arrows) on the right iliopsoas muscle. CT scanning results of the patient before and after the biopsy procedure were compared and it was recognized that the lesion associated with the right ureter, emerging following the biopsy procedure was a lesion in fluid density consistent with urinoma. Urinomas may be asymptomatic and occult initially, but may lead to abscess formation and electrolyte imbalances if not diagnosed and managed appropriately (1). Ureteral leaks can result from trauma, ureteral obstruction, tumors or endourologic interventional procedures (2,3,4). The second most common complication of abdominal laparoscopic surgery is ureteral injury (5). Urinomas are rare complications of ureteroscopy. The diagnosis is usually made by ultrasound or CT (2). The incidental discovery of a urinoma by ^18^F-FDG PET/CT was previously reported (6,7). In our case, urinoma originating from iatrogenic urinary tract injury following endourological biopsy procedure was detected incidentally on PET/CT scan.
